# Research on the relationship between marital commitment, sacrifice behavior and marital quality of military couples

**DOI:** 10.3389/fpsyg.2022.964167

**Published:** 2022-10-04

**Authors:** Lemin Lin, Hang Guo, Lisa Duan, Li He, Chaoliang Wu, Zhangya Lin, Jiangnan Sun

**Affiliations:** ^1^Postgraduate Group, Logistics University of People’s Armed Police Force, Tianjin, China; ^2^Characteristic Medical Center of Chinese People’s Armed Police Force, Institute for Military Psychological Efficacy Evaluation and Stress Intervention, Tianjin, China; ^3^Department of Neurosurgery, The First Affiliated Hospital of Fujian Medical University, Fuzhou, China; ^4^Department of Psychology, The First Affiliated Hospital of Fujian Medical University, Fuzhou, China

**Keywords:** military couples, marital commitment, sacrifice behavior, marital quality, paired samples

## Abstract

Based on the actor-partner interdependence model, this paper studied the relationship between marital commitment, sacrifice behavior and marital quality of military couples. A convenience sample of 171 Chinese military couples from Guangdong, Jiangsu and Sichuan province was used. All participants completed the self-report questionnaires independently including the Dimension of Commitment Inventory (DCI), the Couples Sacrifice Behavior Scale (CSBS) and the Evaluation and Nurturing Relationship Issues, Communication and Happiness (ENRICH). Results showed that the scores of marital commitment and marital quality of male soldiers were significantly higher than that of their spouses. Compared to their spouses, male soldiers reported higher frequency of sacrifice behaviors and perceiving sacrifice behaviors of spouses. Furthermore, the marital commitment of military couples had significant influence on their own marital quality and frequency of perceiving each other’s sacrifice behavior Military couples’ perception of the frequency of each other’s sacrifice behavior partially mediated the effect of marital commitment on their marital quality. Male soldiers’ perception of spouse’s sacrifice behavior frequency significantly predicted the marital quality of their spouses.

## Introduction

Marital relationship is a special interpersonal relationship and social relationship, which is an important aspect of human life. It can also be said that the most fundamental or critical connection between man and woman is marriage, which is a recognized legal, social and ritual (Aman et al., 2019, 2021). Marital quality refers to the attitudes and opinions of individuals toward their spouses and marital relationships (Xu and Ye, 1998).

Different countries and cultures lead to different marital relationships. Study concludes that there are two main differences in spousal relationship between Chinese marriages and Western countries’ marriages, which might cause different marital qualities (Li, 2018). One of the differences is that the unequal status in the Chinese family and equal status in Western countries. Another difference is that Chinese families tend to share economic responsibilities while Western families usually go Dutch. Li (2018) concluded that historical, religious and cultural values, family environmental and other factors could be responsible for bringing the above differences. For example, previous studies have shown that religious factors are correlated with marital commitment and marital satisfaction (Juvan and Dolnicar, 2017; Aman et al., 2019, 2021). Few studies stated that sexual life may indicate the instability of intimate relationships (Zeidabadi et al., 2022). Notably, the unprecedented novel virus COVID-19, which struck more than 200 countries and territories, has led to unexpected crises and changed orders of daily lives (Aqeel et al., 2021; Su et al., 2021; Al Halbusi et al., 2022; Aman et al., 2022; Ge et al., 2022; Geng et al., 2022; Li et al., 2022; Yu et al., 2022; Zeidabadi et al., 2022; Zhou et al., 2022), including people’s marital quality. Therefore, given the importance of participation in marriage life to maintain the mental health of couples (Aman et al., 2021), the study aimed to explore other factors that influence marital quality.

Individuals in military marriages may experience unique challenges that ordinary people do not live through, including deployment, combat stress, war or conflict strikes, lack of emotional expression, military subculture adjustment, long and frequent family separations, frequent moves (Dieryck, 2003), job changes and cultural norms in different countries (National Healthy Marriage Resource Center, 2009). Due to the special characteristics of military occupation and military marriages, the marital quality of military couples has attracted attention. Studies conducted by American psychologists have found that the most common reason of soldiers’ suicide in Iraq was related to the breakdown of their relationships with spouses or lovers (Seligman, 2012). Moreover, if the marital quality of the military personnel is not high enough to provide sufficient support against the devastating consequences (Orthner and Bowen, 1990; Aman et al., 2021), the combat capacity and mission effectiveness of the military could be weaken, thus making the country less safe (National Healthy Marriage Resource Center, 2009). It is possible that healthy marriage and high level of marital quality may contribute to better living quality of military couples that serves the public interest and the country’s safety. Therefore, it is important to study marital quality of military couples.

Research on Chinese military personnel has found that their special occupational characteristics induce marital pressure to them, which may have negative impacts on their marriage (e.g., provoking disputes, escalating conflicts, causing indifference and marital crisis), leading to prominent marital problems and high divorce rate (Feng, 2017; Yang, 2017; Zhu, 2019). Past studies also found that focusing on the negative factors of marriage can hardly play a constructive role in marital quality, while the positive aspects of marriage such as commitment, sacrifice, forgiveness, sanctification and other factors play a repairing role (Hou et al., 2015). In order to gain a further understanding about the marital quality of military couples and bring new insights to the field, Chinese military couples were invited to participate current study.

### Marital commitment and marital quality

Marital commitment is defined as an individual’s desire to remain in a marital relationship, including tendencies of long-term commitment and feelings of psychological attachment (Li et al., 2009; Deniz and Yozgat, 2013). Generally, the quality of marital commitment indicates the level of happiness and satisfaction of the individuals in their marital relationships (Aman et al., 2019). Adams and Jones (1997) proposed a three-dimensional structure of marital commitment, namely, commitment to spouse, commitment to marriage, and the sense of limitation. Xu (2012) believed that the study of marital commitment can help Chinese couples understand the psychological structure of each other, their attitudes toward marriage, and their views on each other, so as to solve their problems scientifically and effectively.

Researches about the relationship between marital quality and commitment have reached inconsistent conclusions. Rusbult (1980) investment model of commitment depicts the relationship between marital quality and commitment, and they may influence each other (Rusbult et al., 2011). Sabatelli and Shehan (2009) found that satisfaction is connected to commitment, whereas commitment extends beyond satisfaction. However, Campbell and Foster (2002) found that the lack of commitment may not be due to satisfaction, which could hint that the link from commitment to satisfaction could be meaningful (Hou et al., 2018). Moreover, commitment is the strongest predictor of persistence in a relationship, accounting for significant variance above and beyond satisfaction, alternatives, and investments (Van Lange et al., 1997; Aman et al., 2021). And the social exchange theory reversed reveals commitment determinates and promotes marital satisfaction (Hou et al., 2018). A good amount of studies has also provided strong evidence for the predictive effect of commitment on marital satisfaction. For example, it was found that higher levels of marital commitment are associated with higher levels of marital satisfaction (Rosen-Grandon et al., 2004). Furthermore, marital commitment is one of the most important factors in satisfactory marriage (Rosen-Grandon et al., 2004). Reversely, lower levels of commitment predict unstable relationship (Rhoades et al., 2010) and divorce (Lavner and Bradbury, 2012). Based on the above evidence, commitment may influence marital quality in different ways. Therefore, the present study chose to examine the relationship between marital commitment and marital quality.

### Sacrifice behavior and marital quality

Sacrifice, from the perspective of intimate relationship, refers to the individual giving up current interests for the needs of his/her partner or the relationship (Cui, 2010; Cao, 2013). Van Lange et al. (1997) described sacrifice as an individual giving up immediate self-interest in order to improve his/her partner’s happiness or relationship quality. According to the interdependence theory (Kelley and Thibaut, 1978) and social exchange theory (Thibaut and Kelley, 1959; Emerson, 1976; Aman et al., 2022), humans are rational beings who seek to maximize rewards and minimize costs in exchanges. Such costs may be negatively correlated with relationship quality (Sabatelli and Shehan, 2009). The intrinsic motivation of sacrifice is that individuals make sacrifices for their partners to anticipate their future sacrifice and reward. At the same time, they can obtain a higher quality relationship through sacrifice (Ma, 2007; Zhai, 2014).

To explore sacrifice’s effect on marital quality, different aspects of sacrifice have been examined, such as frequency of sacrifice (Totenhagen and Curran, 2011), awareness of sacrifice (Akçabozan et al., 2016), perception of sacrifice (Tang et al., 2014; Curran et al., 2015), ease or difficulties of sacrifice (Akçabozan et al., 2016), motivation and behavior of sacrifice (Lan, 2010), perceived cost of sacrifice (Cao et al., 2016; Lan et al., 2017) as well as the perceived inequity of sacrifice (Lan et al., 2017). While researchers have found that frequency of intimate sacrifices was not correlated with any aspects of relationship quality (Curran et al., 2015), other studies have confirmed that how individual perceived the sacrifice in relationship is very important (Akçabozan et al., 2016). Sacrifice theorists also have emphasized that sacrifice needs to be understood in terms of its meanings by both spouses (Whitton et al., 2002; Cao et al., 2016). Moreover, perceived partner’s awareness of intimate sacrifice was correlated with relationship quality (Curran et al., 2015). Referring to previous research, the present study selected the perception of marital sacrifice behavior as one of the variable, which could include the perception of inequity of sacrifice.

Previously, a lot of studies have discussed the relationship between sacrifice behavior and marital commitment. It is believed that the relationship between sacrifice and commitment is complex, and there are mainly three different points of view. Some researchers have found no association between partners’ frequency of sacrifices and individuals’ relationship commitment (Totenhagen et al., 2013). Akçabozan et al. (2016) examined the importance of different aspects of sacrifices (frequency, ease and awareness), in interactions with gender, in understanding variability of commitment for women and men. The commitment they focused on was the daily feelings of commitment across a week. Other researchers suggested that commitment is a central motive in ongoing relationships and proposed that feelings of commitment promote pro-relationship transformation and willingness to sacrifice even when their marriage is not rewarded (Van Lange et al., 1997; Aman et al., 2021). Taken together, the current study aimed to examine how marital commitment affects sacrifice behavior.

Past research have varied conclusions about the relation between sacrifice and relationship quality (Ma, 2007; Zhai, 2014). In general, most findings suggest that sacrifice is detrimental to relationship. For instance, the attachment theory indicates that a high level of sacrifice is harmful to relationships (Bartholomew, 1990). The feminist theoretical model suggests that there are too many sacrifices involving women in relationships (Gilligan, 1977). According to Aman et al. (2019), religious couples have negative feelings about divorce and are willing to sacrifice for each other to maintain their marriage. Thus, the present study planned to further explore the relationship between sacrifice behavior and marital quality.

### The mediation role of sacrifice behavior on marital commitment to marital quality

Previous empirical findings have suggested the possible mediation role of sacrifice behavior on marital commitment to marital quality. Van Lange et al. (1997) have examined the plausibility of a model of willingness to sacrifice based on the principles and constructs of interdependence theory (Thibaut and Kelley, 1959; Kelley and Thibaut, 1978). One of the results reveals that commitment is the strongest predictor of persistence in a relationship, accounting for significant variance above and beyond satisfaction, alternatives, and investments (e.g., Rusbult, 1983; Lund, 1985; Rusbult et al., 1986; Simpson, 1987; Felmlee et al., 1990; Drigotas and Rusbult, 1992). Although their findings cannot form confident causal inferences, their interdependence-based interpretation assumes that willingness to sacrifice partially mediating the link between commitment and functioning. In other words, willingness to sacrifice partially explains the link between commitment and functioning. Moreover, sacrifice may represent one concrete mechanism by which committed individuals are able to develop and sustain healthy, ongoing involvements (Van Lange et al., 1997). Cao et al. (2016) also expected a mediation model of commitment, sacrifice and relationship well-being in their research. They found that marital sacrifice, as a commitment-inspired factor that maintains relationship, functions as a behavioral signal of devotion to the partner and has the potential to promote mutual trust and lead to feelings of satisfaction (Wieselquist et al., 1999; Stanley et al., 2006, 2010).

Results of previous study have demonstrated the possibility that marital commitment is able to enhance the willingness to sacrifice, and it can serve as a predictor of sacrifice (Van Lange et al., 1997; Ma, 2007). Referring to above research, the current study proposed that the perception of sacrifice behavior mediates the relationship between marital commitment and marital quality.

In sum, based on the interdependence theory and empirical evidences from previous literature, several research questions were put forward in the context of military marriage. Firstly, can marital commitment of military marriages predict sacrifice behavior? Secondly, are marital commitment and sacrifice behavior the key factors that affect marital quality of military couples? Can marital commitment affect marital quality of military couples through sacrifice behavior? Therefore, the present study intends to investigate the current situation and characteristics of marital commitment, sacrifice behavior and marital quality of military couples, and to examine the relationship among marital commitment, sacrifice behavior and marital quality of military couples by applying the actor-partner interdependence model.

## Materials and methods

### Research objectives, hypotheses and methodology

**Figure 1:**
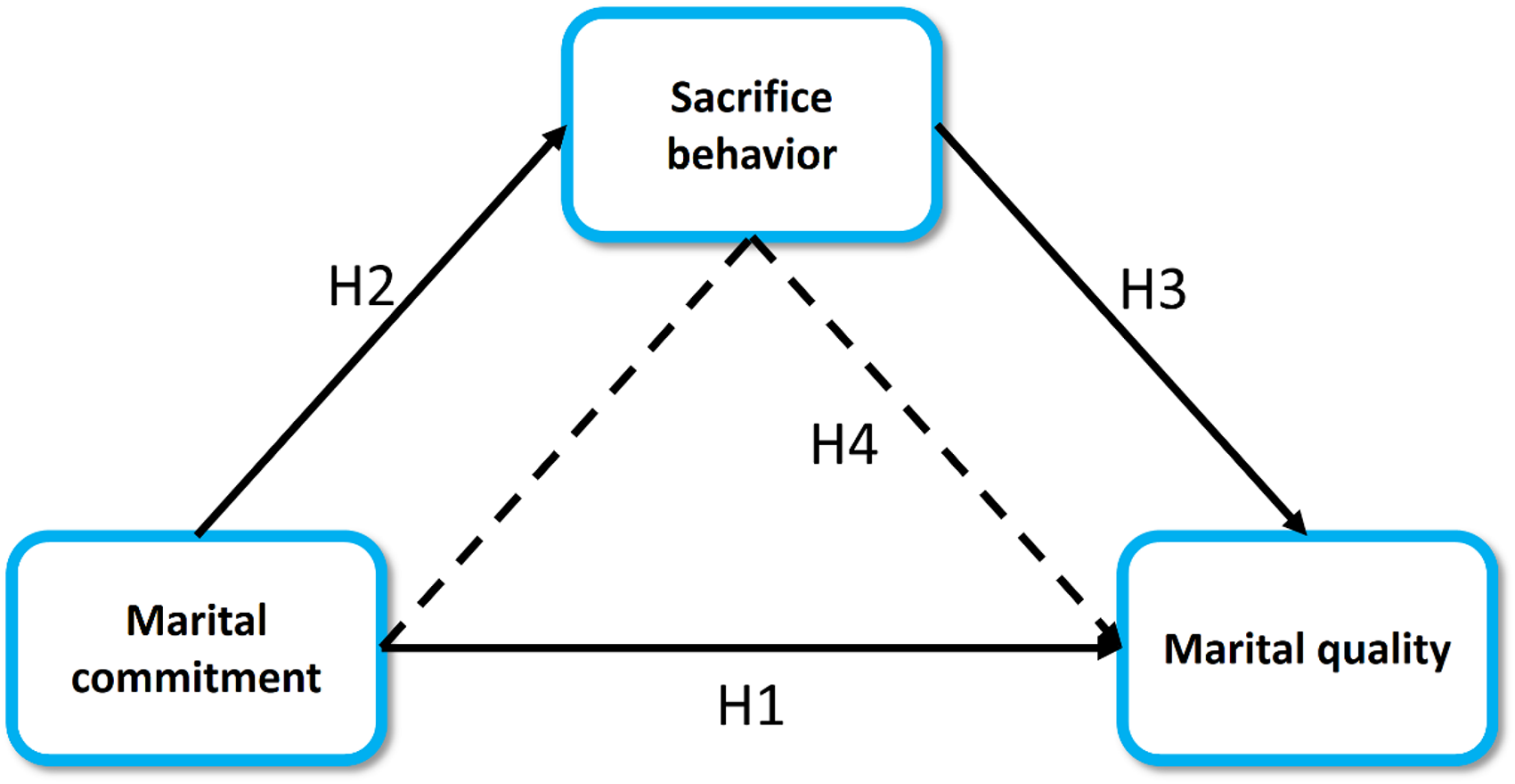
The research framework.

The present study aimed to explore the relationship among marital commitment, sacrifice behavior and marital quality of Chinese military couples (Aqeel et al., 2022). According to the literature review and the proposed research questions, this study formulated the research framework (Figure 1) and the following hypotheses. H1: the marital commitment will significantly influence the marital quality. H2: the marital commitment will positively influence the sacrifice behavior. H3: the sacrifice behavior will significantly correlate with the marital quality. H4: the perception of sacrifice behavior will positively mediate the relationship between marital commitment and marital quality.

Structural equation modeling (SEM) is a robust statistical technique and frequently used in social science which combines regression, multiple correlations, factor analysis and path analysis techniques (Aman et al., 2022; Yu et al., 2022). The SEM method can examine and test complex connections and causal linkages systems. Because latent (unobserved) variables cannot be measured directly using manifest (observable) variables, it is critical to create a model to quantify them (Rahmat et al., 2022; Aman et al., 2022). The path model is employed to test the above hypotheses.

### Research design, sample and data

The purpose of the present study was to explore the relationship among marital commitment, sacrifice behavior and marital quality of Chinese military couples by using a descriptive, observational, cross-sectional and quantitative approach with empirical data (Aqeel et al., 2021; Moradi et al., 2021; Farzadfar et al., 2022; Yao et al., 2022; Yu et al., 2022).

Convenience sampling and snowball sampling methods were used to select married male soldiers and their spouses in Guangdong, Jiangsu and Sichuan provinces as participants of the study. The recruitment was from November 2021 to March 2022. Report indicates that among active-duty soldiers, more than 9 in 10 military spouses are women (National Healthy Marriage Resource Center, 2009). Moreover, the ratio of male to female soldiers in China is close to 19:1. Therefore, the present study considered only male soldiers and their spouses in order to collect data in a timely manner. The sample size was determined based on the inclusion criteria and at least a 95% confidence level with 80% test power (Moradi et al., 2021; Geng et al., 2022; Guo et al., 2022; Yao et al., 2022). The inclusion criteria were: (a) Chinese male soldiers and their female spouses, (b) individuals who were willing to participate in the study. The exclusion criteria applied to all Chinese soldiers who wasn’t in their first marital relationship and those who did not indicate willingness to participate in the study (Yao et al., 2022; Zeidabadi et al., 2022). A total of 400 questionnaires were sent out and 385 were recovered, with a recovery rate of 96.25%. After eliminating 8 questionnaires with missing data, 10 questionnaires with consistent answers and 25 questionnaires that could not be paired with a husband and wife, 342 valid questionnaires were collected. The final sample consisted of 171 military couples who completed the questionnaire entirely and respondents’ response rate was 85.5%. Participants were educated about the purpose of the survey and assured them of data confidentiality.

Both paper and online questionnaires were used. Assistance was offered to those who were having hard time understanding the questions. The questionnaire was written in Chinese and contained two main parts. The first part was the demographic questionnaire. The second part consisted of three subsections, and each subsection contains items measuring the key variables.

#### Demographic questionnaire

The self-designed demographic questionnaire was constructed on the basis of literature review, and the questions involved gender, age, year(s) of marriage, education, working hours and living arrangement.

#### The dimension of commitment inventory

The revised Chinese version of DCI (Li, 2006) was adopted for this study. It consisted of measurements of 3 dimensions, including commitment to spouse, commitment to marriage and feeling of limitation. Each dimension is measured by 15 items. Commitment to spouse referred to a commitment based on loyalty and satisfaction, based on one’s own devotion, love and attachment. Commitment to marriage reflected an individual’s belief in marriage. Feeling of limitation was defined as being constrained by economic and external social pressures and it was a limiting force that prevents individuals from divorcing. Responses were rated on a 5-point Likert scale ranging from 1 (Strongly disagree) to 5 (Strongly agree). The total score of the DCI was the average score of all items. In the present study, the Cronbach’s α of the DCI was.920. The Cronbach’s α of the DCI were.909 and.925 for soldiers and their spouses, respectively. Thus, the scale specifies high internal reliability.

#### Couples sacrifice behavior scale

The revised Chinese CSBS (Lan, 2010) was used to measure sacrifice behavior of couples. It measured sacrifice behavior in 3 dimensions, namely emotional support, action and compromise. Emotional support was the act of temporarily ignoring one’s own likes and dislikes in order to satisfy emotional needs of one’s spouse. Action was defined as efforts made to help a spouse achieve some realistic goals. Compromise was referred to the act of adjusting or changing one’s attitude in order to conform attitudes or perceptions of his/her spouse. Participants rated their own sacrifice behavior in terms of frequency and degree. Subsequently, they also rated their perceived sacrifice behavior of partners in terms of frequency and degree. Responses were rated on a 5-point Likert scale with frequency ranging from 1 (Never) to 5 (Always) and degree ranging from 1 (Not making sacrifice at all) to 5 (Make great sacrifice). This questionnaire did not have a cut-off point. The higher the score, the higher the frequency and degree of sacrifice behavior rated. The total score of the scale was the average score of all items. In the current study, the Cronbach’s α for the CSBS was.988. The Cronbach’s α of CSBS were.988 and.988 for soldiers and their spouses respectively, thus showing high internal reliability.

#### Evaluating and nurturing relationship issues, communication, happiness

The revised Chinese version of ENRICH (Mao, 2019) was used to investigate the degree of marriage satisfaction of participants and identify the conflicts in their marriage. Responses were rated on a 5-point Likert scale ranging from 1 (It is really not true) to 5 (It is really true). This questionnaire did not have a cut-off point. The higher the total scores of all the questions, the better the marital quality (Feng, 2021). The scale consists of 12 factors and can be selected according to research needs. In this study, 10 items were selected as the “marriage satisfaction” factor. The score lower than the norm indicated low levels of marital quality. The score within the norm suggested medium levels of marital quality. If the score was higher than the norm, the levels of marital quality was high (Li and Yang, 1990). In the current study, the Cronbach’s α for ENRICH was.864. The Cronbach’s α of ENRICH were.847 and.874 for soldiers and their spouses, respectively. Therefore, the scale demonstrated good reliability.

### Data analysis plan

The analysis was performed based on the empirical data provided by participants. Cronbach’s α tests were used to measure the internal consistency (reliability) of items within each scale. If the Cronbach’s α was greater than.80, the reliability was very good (Al-Sulaiti et al., 2021; Azadi et al., 2021). Frequency, percentage, mean and standard deviation were applied to describe the demographics of sample. SPSS version 19 was used to investigate the differences of marital commitment, sacrifice behavior and marital quality between military couples through the paired samples t-test. Pearson correlation method was employed to test the relationship between marital commitment, sacrifice behavior frequency and marital quality of military couples. There were two kinds of evaluations. The first one was the evaluation of the measurements of the external model, and the second was the evaluation of the internal structural model (Aman et al., 2022; Yu et al., 2022). AMOS version 26 structural equation model was utilized to examine the relationship among marital commitment, sacrifice behavior and marital quality of military couples. The χ^2^/df < 3.00, RMSEA<0.08, CFI > 0.90, TLI > 0.90, and GFI > 0.90 suggests a good fit (Guo et al., 2022).

## Results

### Common method bias test

As the data in this study came from self-reports of participants, there might exist common method bias. The Harman single factor test was used to examine the possible common method bias. Results showed that 29 factors with characteristic roots greater than 1 were obtained without rotation, and the variation explained by the first factor was 38.8% (<40%). This suggested that the common method bias had no significant influence on the results of this study.

### Descriptions of participants’ demographics

The participants’ age ranged from 20 years to 43 years, with a mean age of 28.9 years (*SD* = 3.1). The mean age of male soldiers was 29.5 years (*SD* = 2.9), and the mean age of their spouses was 28.4 years (*SD* = 3.2). The mean years of current marriage was 2.8 years (range from 0.1 to 13, *SD* = 2.2), and 86.75% of the participants married for no more than 5 years. The detailed demographic information of participants is presented in Table 1.

**Table 1:** Demographic information of participants.

		Total		Soldier		Spouse	
		N	Percentage	N	Percentage	N	Percentage
Age (years)	20–25	47	13.74%	14	4.09%	33	9.65%
	26–30	197	57.60%	98	28.65%	99	28.95%
	31 or above	98	28.65%	59	17.25%	39	11.40%
Number of Years in Current Marriage	0–1	116	33.92%	58	16.96%	58	16.96%
	1–3	118	34.50%	59	17.25%	59	17.25%
	3 or above	108	31.58%	54	15.79%	54	15.79%
Education	High school or below	72	21.05%	39	11.40%	33	9.65%
	College	111	32.46%	57	16.67%	54	15.79%
	Undergraduate or above	159	46.49%	75	21.93%	84	24.56%
Working years	0–5	123	35.96%	17	4.97%	106	30.99%
	6–10	120	35.09%	68	19.88%	52	15.20%
	11 or above	99	28.94%	86	25.14%	13	3.80%
Only child	Yes	258	75.44%	124	36.26%	134	39.18%
	No	84	24.56%	47	13.74%	37	10.82%
Living arrangement	Live together	56	16.37%	28	8.18%	28	8.18%
	Live apart	286	83.63%	143	41.81%	143	41.81%

### Descriptive statistics

Statistical analysis of the military couples’ scores of marital commitment, sacrifice behavior and marital quality suggested that their scores were all at the upper-middle level (Li, 2006; Lan, 2010; Hou et al., 2015; Gu, 2017). The paired sample *t*-test was used to further explore the differences in perceived marital commitment, sacrifice behavior and marital quality between soldiers and their spouses. The results are shown in Table 2. The soldier’s total score of marital quality and total marital commitment scores was significantly higher than that of the spouse (*t _MQ_* = 3.13, *p* < 0.01; *t _MC_* = 3.97, *p* < 0.001).

**Table 2:** Differences and correlations of marital commitment, sacrifice behavior and marital quality of soldiers and their spouses.

	Total		Soldier		Spouse		*t*	Sig.	Correlation	Sig.
	*M*	*SD*	*M*	*SD*	*M*	*SD*				
Marital quality	40.95	7.9	42.03	7.3	39.9	8.28	3.13^**^	0.002	0.332^***^	0
Marital commitment	166.47	20	170.4	19	163	21.4	3.97^***^	0	0.175^*^	0.022
Commitment to spouse	63.11	7.6	63.33	7.2	62.9	8.02	0.64	0.523	0.311^***^	0
Commitment to marriage	57.72	8.6	59.15	7.8	56.3	9.19	3.62^***^	0	0.264^***^	0
Feeling of limitation	45.63	9.7	47.89	8.7	43.4	10.1	4.50^***^	0	0.033	0.668
Self-rated sacrifice behavior	5.82	2	5.93	1.9	5.7	2.03	1.35	0.18	0.332^***^	0
Self-rated frequency of sacrifice behavior	2.9	1	3.01	0.9	2.79	1	2.50^**^	0.013	0.311^***^	0
Self-rated degree of sacrifice behavior	2.92	1	2.93	1	2.91	1.06	0.18	0.858	0.316^***^	0
Self-rated frequency of emotional support	2.86	1	2.86	1	2.87	1.02	−0.11	0.914	0.357^***^	0
Self-rated degree of emotional support	2.89	1.1	2.85	1.1	2.93	1.1	−0.78	0.437	0.323^***^	0
Self-rated frequency of action	3	1	3.19	0.9	2.8	1.02	4.25^***^	0	0.233^**^	0.002
Self-rated degree of action	2.85	1.1	2.87	1.1	2.83	1.09	0.45	0.654	0.373^***^	0
Self-rated frequency of compromise	2.85	1	3	1	2.7	1.07	3.17^**^	0.002	0.243^**^	0.001
Self-rated degree of compromise	3.02	1.1	3.07	1.1	2.97	1.19	0.92	0.361	0.191^*^	0.012
Perceived sacrifice behavior	6.2	2.1	6.39	1.9	6.01	2.22	−1.86	0.064	0.179^*^	0.019
Perceived frequency of sacrifice behavior	3.13	1	3.25	0.9	3.01	1.11	2.37^**^	0.019	0.159^*^	0.038
Perceived degree of sacrifice behavior	3.07	1.1	3.14	1	3	1.14	1.28	0.201	0.204^**^	0.007
Perceived frequency of emotional support	3.16	1	3.29	0.9	3.03	1.1	2.45^***^	0.015	0.128	0.096
Perceived degree of emotional support	3.08	1.1	3.16	1	3	1.13	1.47	0.143	0.209^**^	0.006
Perceived frequency of action	3.15	1.1	3.27	1	3.03	1.14	2.23^*^	0.027	0.152^*^	0.047
Perceived degree of action	3.1	1.1	3.18	1	3.03	1.17	1.36	0.174	0.187^*^	0.014
Perceived frequency of compromise	3.07	1.1	3.19	1	2.96	1.15	2.25^*^	0.026	0.174^*^	0.023
Perceived degree of compromise	3.02	1.1	3.07	1.1	2.97	1.19	0.92	0.361	0.191^*^	0.012

* *p* < 0.05;

** *p* < 0.01;

*** *p* < 0.001.

In terms of sacrifice behavior, there was no significant difference between the scores of self-rated sacrifice behavior and perceived sacrifice behavior of spouse (*t _SSB_* = 1.35, *p* = 0.18; *t _PSB_* = −1.86, *p* = 0.06). The sacrifice behavior frequency of soldiers was significantly higher than that of their spouses (*t* = 2.5, *p* < 0.05), as well as the frequency of action (*t* = 4.25, *p* < 0.001) and the compromise frequency (*t* = 3.17, *p* < 0.01). There was no significant difference in the frequency of emotional support. In regards to the perceived sacrifice behavior of spouse, there was significant difference in the frequency of the sacrifice behavior between soldiers and their spouses (*t* = 2.37, *p* < 0.05). However, there was no significant difference in the degree of sacrifice behavior between soldiers and their spouses. The perceived frequency of emotional support was significantly different between soldiers and their spouses (*t* = 2.45, *p* < 0.05).

Pearson correlation method was used to measure the correlation between marital commitment, sacrifice behavior and marital quality of military couples. As shown in Table 3, the marital quality of soldiers was significantly and positively correlated with their own marital commitment (*p* < 0.01), frequency of sacrifice behavior (*p* < 0.05) and the frequency of perceived sacrifice behavior of spouse (*p* < 0.05). However, the marital quality of soldiers was not significantly correlated with spouses’ marital commitment, frequency of sacrifice behavior and the frequency of perceived sacrifice behavior. The marital quality of soldiers’ spouses was significantly and positively correlated with their own marital commitment (*p* < 0.01), frequency of sacrifice behavior (*p* < 0.05), the frequency of both soldiers (*p* < 0.05) and their spouses (*p* < 0.01) perceived partner’s sacrifice behavior. Nevertheless, the marital quality of soldiers’ spouses was not significant correlated with soldiers’ marital commitment and frequency of sacrifice behavior.

**Table 3:** Correlation among study variables.

	Soldier’s marital quality	Soldier’s marital commitment	Frequency of soldier’s sacrifice behavior	Soldier’s perceived frequency of sacrifice behavior	Marital quality of spouse	Marital commitment of spouse	Sacrifice behavior frequency of spouse
Soldier’s marital quality	–						
Soldier’s marital commitment	0.337^**^	–					
Frequency of soldier’s sacrifice behavior	0.170^*^	0.208^**^	–				
Soldier’s perceived frequency of sacrifice behavior	0.309^**^	0.292^**^	0.734^**^	–			
Marital quality of spouse	0.332^**^	0.06	0.139	0.159^*^	–		
Marital commitment of spouse	0.129	0.175^*^	0.198^**^	0.219^**^	0.443^**^	–	
Sacrifice behavior frequency of spouse	0.033	0.11	0.311^**^	0.219^**^	0.161^*^	0.313^**^	–
Spouse’s perceived sacrifice behavior frequency	0.083	0.05	0.167^*^	0.159^*^	0.302^**^	0.354^**^	0.780^**^

* Significantly correlated at the 0.05 level (two-sided).

** Significantly correlated at the.01 level (two-sided).

Regression analyses were performed to examine the effect of participants’ own marital commitment on the perceived sacrifice behavior frequency and marital quality. The marital commitment of both soldiers and their spouses significantly and positively predicted their own marital quality (soldiers: *β* = 0.337, *ΔR^2^* = 0.108, *p* < 0.001; spouses: *β* = 0.443, *ΔR^2^* = 0.191, *p* < 0.001) and their perceived sacrifice behavior frequency (soldiers: *β* = 0.292, *ΔR^2^* = 0.086, *p* < 0.001; spouses: *β* = 0.354, *ΔR^2^* = 0.125, *p* < 0.001). Moreover, the frequency of soldiers’ perception of their spouses’ sacrifice behavior significantly and positively predicted their own marital quality (*β* = 0.309, *ΔR^2^* = 0.095, *p* < 0.001). The frequency of spouses’ perception of their husbands’ sacrificial behavior significantly and positively predicted their own marital quality (*β* = 0.302, *ΔR^2^* = 0.091, *p* < 0.001).

### Actor-partner interdependence model evaluation

**Figure 2:**
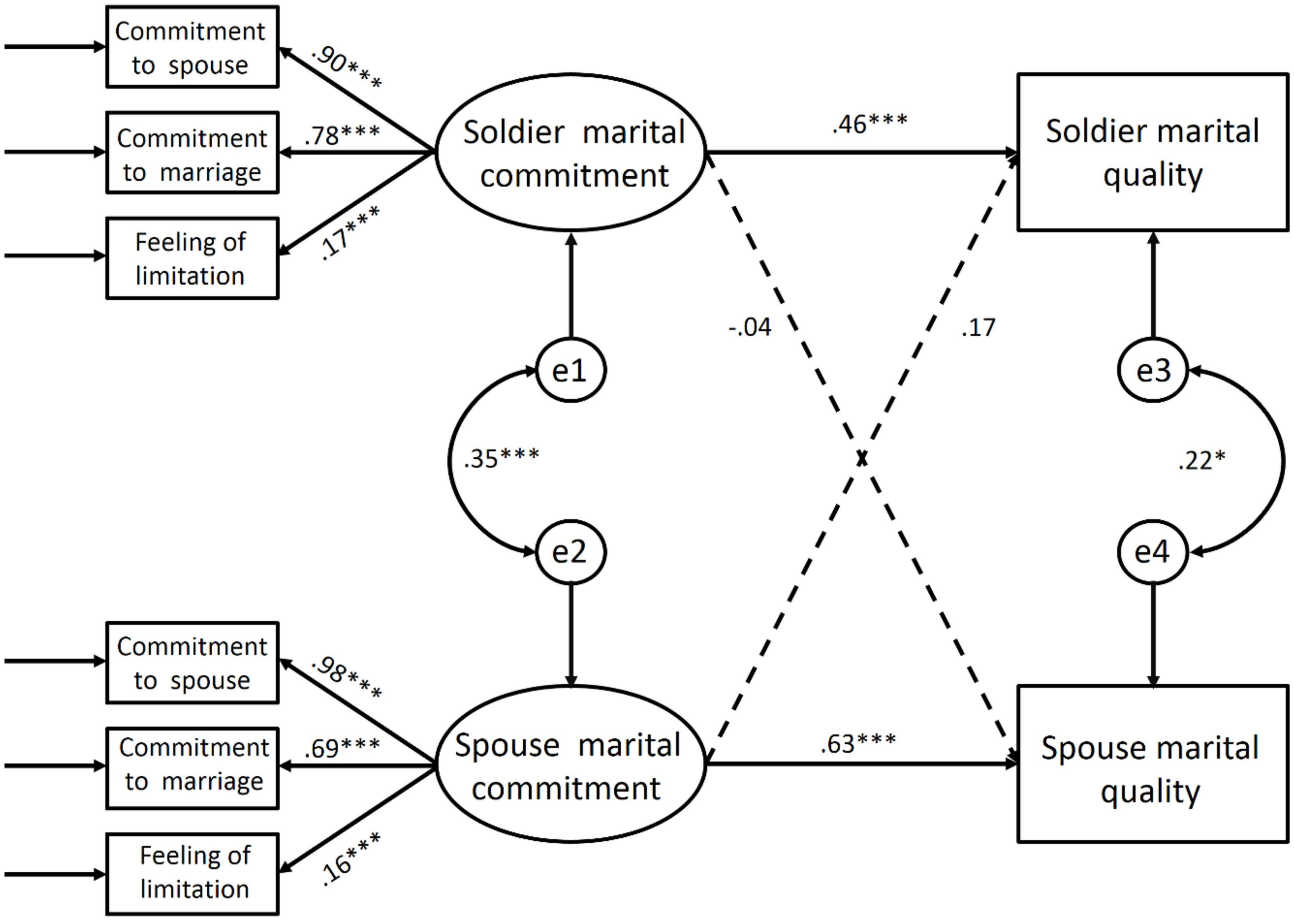
The path relationship between soldier and spouse marital commitment and their respective marital quality. ^*^p < 0.05; ^***^p < 0.001.

To examine the effect of marital commitment of soldiers and their spouses on the marital quality, the data of soldiers and their spouses were combined, the idea of actor-partner interdependence model was adopted and the method of structural equation model was used. Results showed that marital commitment of both soldiers and their spouses positively predicted their own marital quality (soldiers: *β* = 0.46, *p* < 0.001; spouses: *β* = 0.63, *p* < 0.001). However, marital commitment of both soldiers and their spouses failed to predict their partners’ marital quality (see Figure 2).

After conducting stratified and stepwise regression analysis, it was found that participants’ commitment to spouses significantly predicted the marital quality of their spouses (soldiers: *β* = 0.181, *ΔR^2^* = 0.033, *p* < 0.05; spouses: *β* = 0.329, *ΔR^2^* = 0.108, *p* < 0.001). The full model provided a good fit to the data (*χ^2^*/*df* = 2.23, RFI =0.873, NFI =0.937, IFI =0.964, TLI =0.926, CFI = 0.963, RMSEA = 0.085).

**Figure 3:**
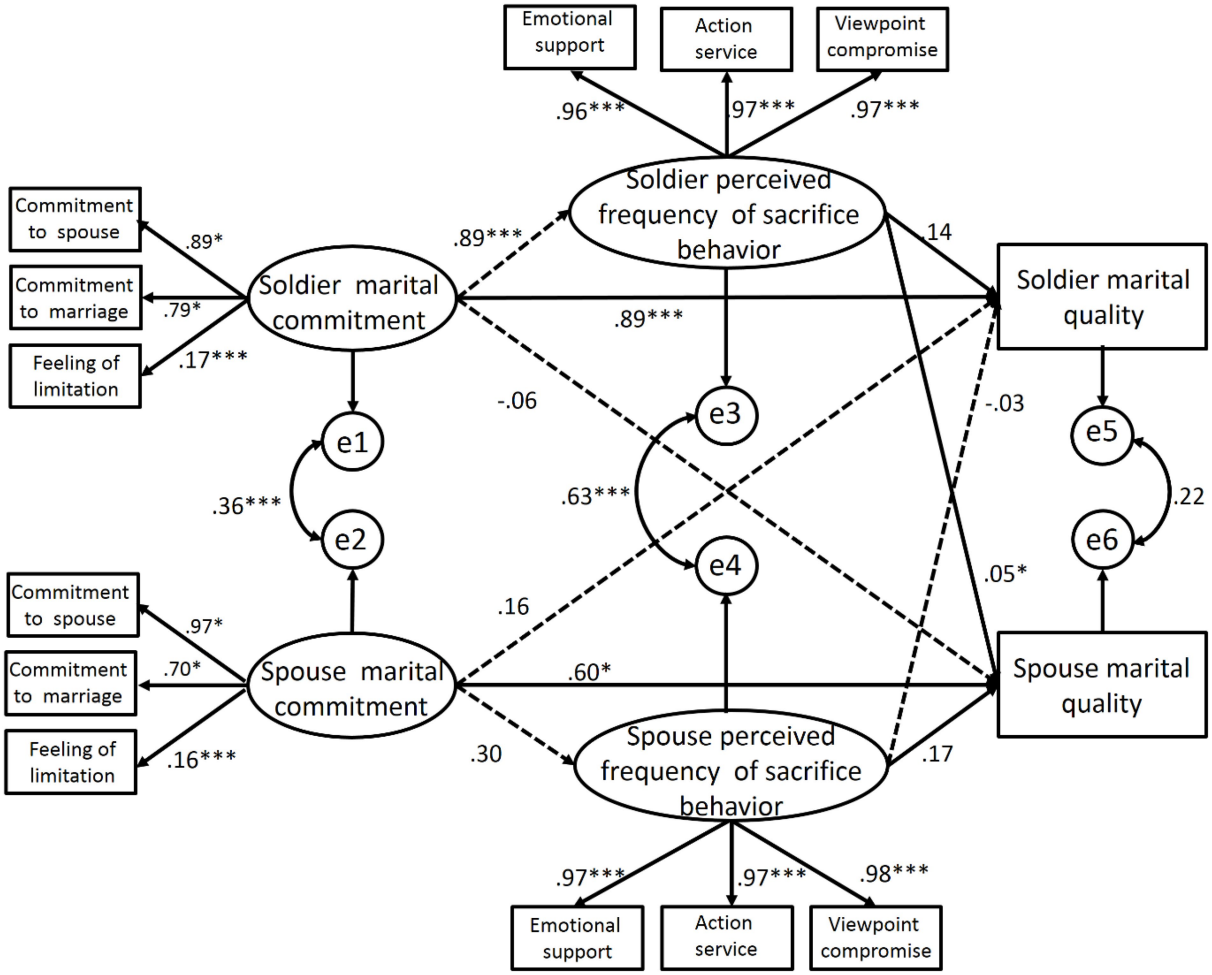
The path of marital commitment, perceived sacrifice behavior frequency and marital quality of military couples. ^*^p < 0.05; ^***^p < 0.001.

How military couples’ perception of each other’s sacrifice behavior frequency affects their own and their partner’s marital quality *via* marital commitment was examined (see Figure 3). Results showed that when the perception of the sacrificial behavior frequency entered the effect path of marital commitment on marital quality, the direct predictive effect of marital commitment on their respective marital quality did not disappear. Partly through the mediating variable, the perceived of sacrifice behavior frequency exerted an indirect effect on their respective marital quality. This suggested that the frequency of sacrifice behavior perceived by military couples played a partial mediating role between their own marital commitment and marital quality. The frequency of sacrifice behaviors perceived by soldiers had a significant predictive effect on the marital quality of their spouses (*p* < 0.05), while the frequency of sacrifice behaviors perceived by their spouses had no significant effect on soldiers’ marital quality. The full model provided a good fit to the data (*χ^2^*/*df* = 1.688, RFI = 0.930, NFI = 0.950, IFI = 0.979, TLI = 0.970, CFI = 0.979, RMSEA = 0.064).

## Discussion

The present study designed a framework based on the theoretical and empirical evidence to investigate the relationship between marital commitment, sacrifice behavior and marital quality among Chinese male soldiers and their spouses. The indirect effect of marital commitment on marital quality through the mediation of sacrifice behavior was examined. The main purpose of the present study was to test the theoretical path model. Results suggests that all the hypotheses were somehow been proven in varying degrees (Lebni et al., 2020; Aqeel et al., 2021). The perceived sacrifice behavior, especially male soldiers’ perception of their spouses’ sacrifice behavior, mediates the effect of marital commitment on marital quality. Although the marital quality of both the male soldiers and their spouses were higher than non-military couples in the present study, there was significant difference between soldiers and spouses in terms of marital quality. The marital quality of spouses was relatively lower than that of male soldiers. The female spouses might not perceive enough sacrifice behavior of husbands as they hope. To better maintain marital quality, it is important that male soldiers care more about the feeling of their spouses and make necessary sacrifice in response to their spouses’ emotional needs. These results are consistent with previous studies (Li, 2006; Lan, 2010; Hou et al., 2015).

### The differences of marital commitment, sacrifice behavior and marital quality between male soldiers and their spouses

Results of the present study found that male soldiers scored higher than their spouses on the score of marital commitment, commitment to marriage and feeling of limitations, and this gender asymmetry of relationship commitment has been found in many societies (Fincham et al., 2007). The marital commitment score of soldiers in this study was higher than the norm score (Li, 2006). It is possible that the core values of the military promote high levels of loyalty of soldiers. Besides, the advent of the COVID-19 posed social, environmental, financial, and mental health challenges worldwide. It has given rise to mobility bans, travel restriction challenges and community lock-downs. However, the consequences of the pandemic may have little effect on military couples compared to ordinary couples (Aqeel et al., 2021, 2022; Al Halbusi et al., 2022; Aman et al., 2022; Ge et al., 2022; Geng et al., 2022; Li et al., 2022; Yu et al., 2022; Zeidabadi et al., 2022; Zhou et al., 2022). It is because that military couples may be more used to lock-downs and living apart in long-distance for a long period of time.

Nevertheless, no significant difference was found in “commitment to spouse” scores between soldiers and their spouses, which was inconsistent with previous research results (Li et al., 2009; Hou and Fang, 2015). It is possible that military couples infrequently spend time together and mostly live apart, and both soldiers and their spouses attach importance to their partners. Therefore, they are willing to take responsibility to live up to their promise to their partners. Soldiers shoulder heavy tasks in the military and they barely have time to take care of their family. At the same time, they have strong desire to maintain a stable marriage and thus keep their commitment to their marriage. In regards to the “feeling of limitations” dimension, soldiers are not only constrained by daily economic and social pressures but also influenced by military discipline. The military’s strict requirements, the society’s reverence, and the legal protection of military marriages and other special professional cultures have exerted pressures on military couples. Coupled with the society’s praise, expectations, and inherent impressions of military wives, it is understandable that military couples score higher than the norm score in the measurement of feeling of limitations.

In the analysis of the frequency of sacrifice behavior of military couples, it was found that soldiers scored lower than their spouses in the self-assessment of emotional support while they scored higher in the self-assessment of other dimensions of sacrificial behavior. Although the difference in emotional support was not significant, it is suspected that soldiers may pay more attention to doing some specific things for their families but somehow they ignore the emotional investment for their spouses and families. Soldiers scored significantly higher than their spouses on the frequency of sacrificial behaviors, likely because soldiers tended to compensate their spouses through behavior during the extremely brief time they spent with their spouses. In the dimension of emotional support, there was no significant difference in self-perception, but there exists significant difference in the perception of their partners. This means that even though the emotional support has been recognized by both sides, soldiers’ emotional support for spouses falls short of their spouses’ expectations. This is in line with the social view theory of marriage that individuals in marriage may focus on duty or emotion (Pan, 1998). Soldiers may be strongly willing to keep commitments that entail a long term-view (Whitton et al., 2007). In other words, they are more traditional and focus on obligations and fulfilling family responsibilities. On the other hand, women are relatively modern and long for romantic love (Cao et al., 2016), so they pay more attention to emotional support and provide more emotional support. Women’s and their emotional well-being might be more affected compared to men because of domestic duties and household chores and complicated works (Lebni et al., 2020). It is likely that the spouse invested more emotional support in military marriage. This also explains why soldiers perceive a higher frequency of emotional support, action and compromise of their spouses. It also reflects that soldiers and their spouses are still influenced by the traditional Chinese marriage culture where husband/male is expected to shoulder financial responsibilities and support the family, and wife/female is responsible for doing housework. Spouses of soldiers possibly do give more in every way and are the major contributors in their marriages. These finding are consistent with studies on functional differences in marital roles (Hou et al., 2015; Hou and Fang, 2015).

Correspondingly, soldiers’ perception of their spouses’ sacrifice behavior frequency was significantly and positively correlated with their spouses’ perception of soldiers’ sacrifice behavior frequency, which is consistent with previous research results (Lan, 2010; Hou et al., 2015). Soldiers and spouses both perceive the other’s sacrifice behavior as more frequent than their own, and tend to give more in return. According to the social exchange theory and the behavioral model theory (Qin, 2018), individuals can feel the sacrifice behavior of their spouses and subsequently form a positive cognition. They are likely to make sacrifice in return, and such interaction will have positive influence on each other. This will be conducive to promoting their marital quality. It could be one of the reasons that the marital quality of military couples were all at the upper-middle level (Li, 2006; Lan, 2010; Hou et al., 2015; Gu, 2017).

It was also found that the marital quality of soldiers and spouses was higher than the norm meanwhile the marital quality of soldiers was significantly higher than that of their spouses. This result is consistent with previous finding (Hou et al., 2015), indicating that the role differences in marital quality also exists in military couples. It is suspected that most military couples face long-term separation, and soldiers’ actual devotion to family is far less than that of their spouse. Therefore, the family pressure is mainly borne by their spouses. As a result, the marital quality of soldiers’ spouses is worse than that of soldiers. Compared to soldiers, their spouses have higher expectation on marriage, and thus have greater psychological gap and disappointment. In addition, the perseverance of soldiers will make them easier to stick with their feelings. They are more active in the perception of marital quality and they are more likely to maintain a stable marital relationship. This result is consistent with Liu’s investigation of police groups (Liu et al., 2020).

### The effect of marital commitment on marital quality

Moreover, results show that the marital quality of soldiers and their spouses was directly affected by their respective marital commitments, meaning that the subjective effect was significant. The higher the marital commitment of the soldiers and their spouses, the higher their marital qualities. The marital quality of soldiers and their spouses was significantly and positively correlated with two dimensions of marital commitment, namely “commitment to spouse” and “commitment to marriage.” This finding is consistent with previous research results (Li, 2006). Past studies have demonstrated that marital commitment is a key determinant of marital quality (Hou et al., 2015), the higher the marital commitment, the more likely individuals are to actively communicate, deal with and solve problems (Rusbult et al., 1998; Li, 2006). Thus, military couples may experience higher marital quality than ordinary couples.

Investigating the objective effect of marital commitment of military couples on marital quality, it was found that when the total score of marital commitment was used to test, there was no objective effect for both soldiers and their spouses. However, when the dimension of “commitment to spouse” in marital commitment was used to test, it was found that there was an objective effect of both the soldiers and their spouses. It means that the marital quality of military couples is not only affected by their “commitment to spouse,” but also influenced by their partners’ “commitment to spouse.” This is inconsistent with results of previous studies where no significant objective effect of husband’s marital commitment was found (Xu, 2012). It is suspected that the “feeling of limitation” in marital commitment may weaken the individual’s perception of marital quality, which is negatively correlated with marital quality. Due to the special nature of the soldier’s work, they spend very limited time on taking care of their families and use more verbal commitments to make the spouse feel their willingness to maintain the marriage in the long term, which may affect the marital quality of their spouses.

### The mediating role of sacrifice behavior in marital commitment and marital quality

From the results of the structural equation model, it was found that the effect of military couples’ marital commitment on marital quality did not completely disappear after adding the perception of the sacrifice behavior frequency of their partner to the model. In other words, the frequency of sacrifice behavior perceived by military couples played a partial mediating role in marital commitment and marital quality, and the subjective effect was still significant. However, unlike marital commitment, the objective effect only existed among soldiers. This suggests that the marital quality of soldiers’ spouses was not only affected by the frequency of self-assessment sacrifice behaviors of soldiers, but also affected by soldiers’ perception of their spouses’ sacrifice behavior frequency. That is, the soldiers’ perception of spouses’ sacrifice behavior frequency had a significant objective effect on the marital quality of their spouses, which confirms that the sacrifice behavior between husband and wife is very helpful for the development of gratitude (Hou et al., 2015). At the same time, the difference in subject-object effect is consistent with the previous research results, and it is still affected by the difference in marital role function. Because the soldiers’ perceptions of their spouse’s sacrifice behavior frequency is actually related to the spouse’s sacrifice behavior frequency, which supports that marital commitment can increase sacrifice behavior (Li, 2006). The sacrifice behavior frequency of soldiers was not significantly correlated with the marital quality of their spouses, while perceiving the sacrifice behavior frequency of their partner is conducive to strengthen the feeling their own marital quality (Lan, 2010).

In addition, the findings imply that there still exist role differences in the meaning of individual sacrifice behavior in military marriages, and the sacrifice behavior of soldiers’ spouses is not entirely beneficial to their marital quality. Past research has pointed out that an individual’s true desire to sacrifice himself/herself in a relationship is significantly associated with an increase in psychological stress and a decrease in relationship satisfaction (Lan, 2010). Feminism believes that sacrifice can lead to marital dissatisfaction and depression, which has a negative impact on marital quality and individual’s mental health, especially for females (Cui, 2010). Therefore, improving the marital quality of military couples should not be achieved by the sacrifice of female spouses.

Results of different mediation paths of the subject-object effects of military couples are also worth noting. It revealed that spouses’ perception of soldiers’ sacrifice behaviors frequency has a limited impact on spouses’ marital quality. If soldiers make commitment without letting their spouses feel the corresponding marital sacrifice behavior, it will be difficult to promote their marriage quality. This also makes it clear that the improvement of military couples’ marital quality should focus on relevant key factors. For instance, the sacrifice behavior of both sides in military marriage is very important. It is essential to let spouses of soldiers to perceive more sacrifice behavior of the soldier in their marriage. The sacrifice of the soldiers could be more crucial to improve the military marital quality. Although soldiers usually make commitments and sacrifice in their marriage, how to better make their spouses perceive their sacrifice behaviors will be a focus of future research.

## Conclusion

The marital commitment of military couples had significant influence on their own marital quality and perception of the frequency of each other’s sacrifice behavior. Military couples’ perception of each other’s sacrifice behavior frequency partially mediated the effect of their own marital commitment on their own marital quality. At the same time, soldiers’ perception of their spouses’ sacrifice behavior frequency significantly predicted the marital quality of their spouses, while the spouses’ perception of soldiers’ sacrifice behavior frequency did not significantly predict the marital quality of soldiers.

### Limitations and recommendations

There are several shortcomings in this study. Firstly, the study was a cross-sectional study that recruited a limited number of participants during a short period of time. It explored the possible mode of action among military couples rather than determining causality. The further research should add longitudinal tracking studies, use larger sample size, employ quantitative research method, try the Strengths, Weaknesses, Opportunities, and Threats (SWOT) analysis method, thematic analysis method (Abaalzamat et al., 2021) or the alternating conditional expectation (ACE) analysis method (Ismail et al., 2009), even incorporate the mixed methods approach to better understand the dynamic process (Aman et al., 2022; Yu et al., 2022). Secondly, the questionnaire did not collect sufficient demographic information that might affect marital quality. For example, the amount of time of being apart due to military service, financial issues, religious beliefs, ethnicity and so on. In order to get deeper insights into the military marital quality, future studies should be more carefully and rigorous about the demographics factors. Moreover, the study only analyzed and discussed the deployed husband but not the deployed wife. Further research should also examine and discuss the difference between the deployed husbands and deployed wives, and consider including control variables like the perceived equity in sacrifice behavior frequency. Last but not least, the present study only selected male soldiers from three provinces of China, which made the sample lack of representativeness. It will be difficult to generalize the results to a broader population of Chinese military couples. A more systematic sampling method should be considered in the future.

In terms of policy implications, the findings of current study suggest that marital quality of military marriage could be potentially improved by raising awareness of increasing sacrifice behavior of military couples. This can be achieved through giving educational lectures, organizing relevant awareness campaigns and providing guidance from marriage counselors. Policymakers need to pay more attention to providing professional psychological support to help military couples manage crises and challenge events in their marital relationships.

## Data availability statement

The raw data supporting the conclusions of this article will be made available by the authors, without undue reservation.

## Ethics statement

Ethical review and approval were not required for the study on human participants in accordance with the local legislation and institutional requirements. Written informed consent from the (patients/participants or patients/participants legal guardian/next of kin) was not required to participate in this study in accordance with the national legislation and the institutional requirements.

## Author contributions

JS and ZL conceived and designed this study. LD, LH, and CW performed the collection of data. LL performed data analysis and visualization. HG drafted and revised the manuscript. JS supervised the whole study. All authors contributed to the article and approved the submitted version.

## Funding

Funding for this research was supported by the scientific research innovation team project of the Characteristic Medical Center of Chinese People’s Armed Police Force (NO. KYCXTD0401) and Special Health Fund of Fujian Provincial Department of Finance (BPB-LZY2019, BPB-LZY2020, and BPB-LZY2021).

## Conflict of interest

The authors declare that the research was conducted in the absence of any commercial or financial relationships that could be construed as a potential conflict of interest.

## Publisher’s note

All claims expressed in this article are solely those of the authors and do not necessarily represent those of their affiliated organizations, or those of the publisher, the editors and the reviewers. Any product that may be evaluated in this article, or claim that may be made by its manufacturer, is not guaranteed or endorsed by the publisher.
