# Supplementary material for: Research on the relationship between marital commitment, sacrifice behavior and marital quality of military couples
Source: Front Psychol. 2022 Oct 4;13:964167. doi: 10.3389/fpsyg.2022.964167 (PMC9577325; doi:10.3389/fpsyg.2022.964167)
Supplement: Supplementary file 1 [file Image_1.pdf]

frontiers

Supplementary Material

1 Supplementary Figures

您好！非常感谢您协助我们进行此项科学研究。这是一份有关婚姻状况的调查问卷，请您根据每个问卷的具体要求，结合自身的实际情况选择一个最符合的答案，并在相应的位置画“√”或填上。调查以**匿名**方式进行，**问卷内所有的问题均无对错之分**，请按您的真实情况回答即可。为了研究的科学性，请您独自完成这份问卷，**不要与您的配偶商讨，**并按照指导语回答所有的问题，尽量**不要有所遗漏。**

回答这份问卷，虽然会有助于进一步提升您的婚姻生活质量，但仍会耽误您一些宝贵的时间（约20分钟），为此我们深表歉意！并再次感谢您的参与，祝您身体健康，生活愉快，工作顺利！

1. 性别：☐男 ☐女
2. 年龄：___岁（整数即可）
3. 婚龄（已结婚时间）：___年（可以是小数，如1.5年）
4. 学历：☐初中及以下 ☐高中 ☐大专 ☐本科 ☐硕士 ☐博士及以上
5. 您已工作的时间：☐0-5年 ☐6-10年 ☐11-15年 ☐16年及以上
6. 您是否独生子女：☐是 ☐否
7. 大部分时间夫妻的居住情况：☐同居 ☐同城异地 ☐完全异地

Supplementary Figure 1. Demographic Questionnaire

问卷一

指导语：婚姻中常常存在相互的承诺和实际的限制，下面45个句子模拟了夫妻在婚姻关系中可能出现的一些情境，请认真阅读每一句，根据您的实际情况在每个句子后最符合您的情况上画“√”。答案无好坏对错之分。谢谢！

项　目	极不同意	不同意	不同意也不反对	同意	极同意
1我尽力让我的婚姻关系美满。					
2无论发生什么事，我的配偶知道我会一直陪在他(她)的身边。					
3我完全忠于我的配偶。					
4当婚姻出现问题时，我会考虑离婚。					
5我愿意为配偶牺牲一切。					
6我希望和配偶白头偕老。					
7如果配偶和我离婚，我会很受伤害。					
8我把配偶和自己看作是一个不可分割的整体。					
9当我想象未来的生活时，总是设想配偶是和我在一起的。					
10我经常幻想和别人结婚会怎样。					
11我对配偶奉献的不多。					
12我认为我和配偶之间有太多无法调和的矛盾。					
13我不太确信我的婚姻会持久。					
14我经常会想和别人在一起的关系会很浪漫。					
15我的未来计划中不包括我的配偶。					
16和配偶离婚是不道德的。					
17婚姻是一生一世的事。					
18我深信夫妻应该彼此忠于对方，无论顺境还是逆境。					
19我觉得没有义务继续维持婚姻关系。					
20我不会离婚，因为这有违我的信仰。					
21我相信婚姻是神圣的。					

Supplementary Figure 2. The Dimension of Commitment Inventory (DCI)

22应该不惜一切代价保护婚姻。					
23如果婚姻存在的问题太多，离婚比较好。					
24婚姻关系决不应该破裂。					
25我很容易想象到一些婚姻破裂的情形。					
26当我和配偶发誓“白头偕老”时，我们知道这意味着永远。					
27与配偶离婚不是一种道德的错误。					
28我认为婚姻不是永恒的。					
29我的配偶和我结婚是因为我们重视婚姻制度。					
30我认为不管发生什么事情，婚姻是一生不变的。					
31离婚会毁了我的名声。					
32我必须和配偶维持婚姻关系,否则我的家人会认为我不好。					
33我的观念是一个人一旦结婚就不应该离婚，无论对婚姻多不满意。					
34如果我离婚，将会是一件不光彩的事。					
35即使我想离开配偶，实际上也是不可能的。					
36离婚不会使我难堪。					
37如果我和配偶离婚，我的家人和孩子会强烈反对的。					
38我为婚姻花了大量的钱财，我决不会和他(她)离婚。					
39我的朋友们不赞成我离婚。					
40如果我很渴望离婚，我会觉得离婚是一种解脱。					
41我不能忍受离婚的耻辱。					
42如果我和配偶分居或离婚，会很没面子。					
43我对他(她)付出那么多，我永远都不会离开他(她)。					
44我害怕如果我离开配偶的话，会遭报应的。					
45如果配偶和我离婚的话，我的家人和孩子会特别难以接受。					

Supplementary Figure 3. The Dimension of Commitment Inventory (DCI)

问卷二

指导语：婚姻生活中面临很多选择，代表了相应的付出频率和程度，以下35个条目模拟了生活中可能的需要付出的情况，请认真阅读每一个句子，根据自己的真实情况，做出两个选择。选择1：在过去一年中，你做出这项行为的频率是怎样的；选择2：这项行为对你而言意味着是多大的付出。答案无好坏对错之分。谢谢！

项目	频率					付出的程度				
	从未出现	很少	有时候	经常	总是	根本不是付出	较小的付出	中等付出	较大的付出	非常大的付出
1我应爱人的要求参加他/她的社交活动或与他/她的家人相聚。										
2我为了跟爱人一起而取消或改变自己原有的计划。										
3我参与爱人感兴趣的活动，即使我自己不感兴趣。										
4为了陪爱人一起，我放弃了自己感兴趣的活动。										
5我帮我爱人的忙。										
6为了帮助爱人，我处理家务，或听从他/她差遣。										
7为了陪爱人一起，我放弃了做自己的工作或做家务。										
8我帮助爱人做他/她的工作或做家务。										
9在我爱人生病或不舒服的时候，我牺牲自己的时间照顾他/她。										
10我为了维护爱人的健康、或促使他/她更健康而付出。(如:为他做有利于健康的饮食，不在她面前吸烟等等)										
11我应爱人的意愿，与他/她的家人呆在一起。										
12我为了陪爱人一起，而缩短了陪我家人的时间。										
13即使爱人在谈论一些我不感兴趣的事情，我也还是注意听。										

Supplementary Figure 4. Couples Sacrifice Behavior Scale (CSBS) (self-assessment)

14我会以我爱人的而不是我自己的方式做事，为了让他/她高兴。										
15我为爱人买礼物。										
16为了爱人，我改变自己原有的消费习惯。										
17我改变自己与异性交往的方式，以取悦我的爱人。										
18我为了让爱人满意而改变自己的外观(如: 发型、衣着等)。										
19我为了取悦爱人而与他/她过性生活。										
20当我的爱人不想过性生活时，我不再勉强他/她。										
21我对我的爱人表达爱意，以取悦他/她。										
22即使很难，我还是会满足我爱人提出的要求。										
23我应我爱人的要求为别人做了些事(如:为孩子、他/她的家人或邻居)。										
24在做决定有不一致的时候，我会妥协，以成全我爱人。										
25我放弃了对事情的控制，而是让我的爱人说了算。										
26我改变自己去适应爱人的生活、卫生等习惯。										
27我为了适应爱人的生活习惯而牺牲自己的健康。										
28我为了与爱人一致，而改变自己对事物的看法。										
29我为了爱人，中断、或调整自己原有的事业发展。										
30无论我多么想说，当爱人不愿听时，我就不再说。										
31我应爱人的要求，减少与自己的一些朋友的接触。										
32我把我们都喜好的东西让给爱人。										
33我承担我们都不喜欢做的事。										
34我为了配合爱人的工作或休息，而调整自己在家的行为。										
35我为了爱人而调整自己的表达方式。										

Supplementary Figure 5. Couples Sacrifice Behavior Scale (CSBS) (self-assessment)

问卷三

指导语：婚姻生活中很多选择，代表了相应的付出频率和程度，以下35个条目模拟了生活中可能的需要付出的情况，请认真阅读每一个句子，请根据您感受到的您爱人的情况也做出选择。答案没有对错之分。同样无需过多思考，只是根据自己印象中的真实情况，做出两个选择。选择1：在过去一年中，你感受到的、你爱人做出以下行为的频率是怎样的；选择2：你觉得这项行为对于你爱人而言，是多大程度的付出。答案无好坏对错之分。谢谢！

项　目	自己感知到的他/她做该行为的频率					该行为对他/她而言付出的程度				
	从未出现	很少	有时候	经常	总是	根本不是付出	较小的付出	中等付出	较大的付出	非常大的付出
1我爱人应我的要求参加我的社交活动，或与我的家人相聚。										
2我爱人为了跟我一起而取消或改变自己原有的计划。										
3我爱人参与我感兴趣的活动，即使他/她自己不感兴趣。										
4为了陪我一起，我爱人放弃了自己感兴趣的活动。										
5我爱人帮我的忙。										
6为了帮助我，我爱人处理家务，或听从我差遣。										
7为了陪我一起，我爱人放弃了做自己工作或做家务。										
8我爱人帮助我做我的工作或做家务。										
9在我生病或不舒服的时候，我爱人牺牲自己的时间照顾我。										
10我爱人为了维护我的健康、或促使我更健康而付出。(如:为我做有利于健康的饮食，不在我面前吸烟等等)										
11我爱人应我的意愿，与我的家人呆在一起。										
12我爱人为了陪我一起，而缩短了陪他/她家人的时间。										
13即使我在谈论一些我爱人不感兴趣的事情，他/她还是注意听。										

Supplementary Figure 6. Couples Sacrifice Behavior Scale (CSBS) (assessment of spouse)

14我爱人会以我的而不是他/她自己的方式做事，为了让我高兴。										
15我爱人为我买礼物。										
16为了我，我爱人改变他/她自己原有的消费习惯。										
17我爱人改变他/她自己与异性交往的方式，以取悦我。										
18我爱人为了让我满意而改变自己的外观(如: 发型、衣着等)。										
19我爱人会为了取悦我而与我过性生活。										
20当我不想过性生活时，我爱人不再勉强我。										
21我爱人对我表达爱意，以取悦我。										
22即使很难，我爱人还是会满足我提出的要求。										
23我爱人应我的要求为别人做了些事(如:为孩子、他/她的家人或邻居)。										
24在做决定有不一致的时候，我爱人会妥协，以成全我。										
25我爱人放弃了对事情的控制，而是让我说了算。										
26我爱人改变自己来适应我的生活、卫生等习惯。										
27我爱人为了适应我的生活习惯而牺牲自己的健康。										
28我爱人为了与我一致，而改变自己对事物的看法。										
29我爱人为了我，中断、或调整自己原有的事业发展。										
30无论我爱人多么想说，当我不愿听时，他/她就不再说。										
31我爱人应我的要求，减少与自己的一-些朋友的接触。										
32我爱人把我们都喜好的东西让给我。										
33我爱人承担我们都不喜欢做的事。										
34我爱人为了配合我的工作或休息，而调整自己在家的行为。										
35我爱人为了我而调整自己的表达方式。										

Supplementary Figure 7. Couples Sacrifice Behavior Scale (CSBS) (assessment of spouse)

问卷四

指导语：婚姻生活是一个充满感受和体验的过程，以下10个句子描述了您在婚姻中的一些感受，请根据您的实际情况在每个句子后最符合你的相应项目上画“√”。希望您能如实填写，不要征求他人的意见，独立完成，条目中的“我们”，均指您和您的配偶。答案无好坏对错之分。谢谢！

项　目	确实不是这样	可能不是这样	不同意也不反对	可能是这样	确实是这样
1我不喜欢配偶的性格和个人习惯。					
2我非常满意夫妻双方在婚姻中承担的责任。					
3我不满意夫妻间的交流，我配偶并不理解我。					
4我非常满意我们作决定和解决冲突的方式。					
5我不满意我们的经济地位和决定经济事务的方法。					
6我非常满意我们的业余活动和夫妻一起度过的时间。					
7对于我们夫妻之间怎样表达情感与性有关的事，我很满意。					
8对于承担做父母的责任分工上，我不满意。					
9我不满意我们与双方父母、朋友的关系。					
10对于我们的价值观，我觉得很好。					

Supplementary Figure 8. Evaluating and Nurturing Relationship Issues, Communication, Happiness (ENRICH)
