# Supplementary material for: Research on the relationship between marital commitment, sacrifice behavior and marital quality of military couples
Source: Front Psychol. 2022 Oct 4;13:964167. doi: 10.3389/fpsyg.2022.964167 (PMC9577325; doi:10.3389/fpsyg.2022.964167)
Supplement: Supplementary file 2 [file Image_2.pdf]

frontiers

Supplementary Material

1 Supplementary Figures

Statement: The English version was translated by the authors and has not been verified for reliability and validity.

Dear. Mr. /Mrs., thank you very much for helping us with this scientific study. This is a questionnaire about marital relationship. Please choose the most suitable answer according to the specific requirements of each questionnaire and your actual situation, and tick "√" or fill in the corresponding position. The survey is conducted anonymously. All the answers in the questionnaire are no right or wrong. For the scientific nature of the research, please complete this questionnaire by yourself, without discussing it with your spouse, and answer all the questions according to the instructions. Please try not to miss anything.

Although answering this questionnaire may help to further improve the quality of your married life, it will still take some of your precious time (about 20 minutes), and we apologize for this! Thank you again for your participation. Best Wishes!

1. Gender: ☐ male ☐ female
2. Age:_____Years (integer is fine)
3. Years of current marriage:_______years (can be a decimal, such as 1.5 years)
4. Education: ☐Junior high school or below ☐High school ☐College ☐Undergraduate ☐Master ☐Doctor or above
5. Working years: ☐0-5 ☐6-10 ☐11-15 ☐16 or above
6. Only child: ☐Yes ☐No
7. Living arrangement: ☐ Cohabitation ☐ Different places in the same city ☐ Completely different places

Supplementary Figure 1. Demographic Questionnaire

Questionnaire 1

Instructions: There are often mutual commitments and practical limitations in marriage. The following 45 sentences simulate some situations that couples may encounter in their marriage relationship. Please read each sentence carefully, tick "√"according to your actual situation. There is no right or wrong answer. Thanks!

Items	Strongly disagree	Disagree	Neither Disagree Nor Agree	Agree	Strongly agree
1. I'm dedicated to making my marriage as fulfilling as it can be.					
2. No matter what, my spouse knows that I'll always be there for him or her.					
3. I am completely devoted to my spouse.					
4. When things go wrong in my marriage, I consider getting a divorce.					
5There is nothing that I wouldn't sacrifice for my spouse.					
6 I want to grow old with my spouse.					
7I would be shattered if my spouse and I divorced.					
8I like knowing that my spouse and I form an inseparable unit.					
9When I image what my life will be like in the future, I always see my spouse standing next to me.					
10I frequently daydream about what it would be like to be married to someone other than my spouse.					
11 I'm not very devoted to my spouse.					
12 I often think that my spouse and I have too many irreconcilable differences.					
13 I am not confident that my marriage will last forever.					
14 I often think about what it would be like to be romantically involved with someone other than my spouse.					
15 My future plans do not include my spouse.					
16 It is morally wrong to divorce your spouse.					
17 Marriages are supposed to last forever.					

Supplementary Figure 2. The Dimension of Commitment Inventory (DCI)

18 I truly believe that spouses should remain devoted to one another "for better or for worse."					
19 I don't feel obligated to remain married to my spouse.					
20 I could never leave .my spouse because it would go against everything I believe in.					
21 I believe in the sanctity, of marriage.					
22 A marriage should be protected at all costs.					
23 If there are too many problems in a marriage, it's okay to get a divorce.					
24 Under no circumstances should the marriage bond be broken.					
25 I can imagine several situations in which the marriage bond should be broken.					
26 When my spouse and I promised "to have and to hold," we knew that it meant forever.					
27 I don't think it's morally wrong to divorce your spouse.					
28 I don't believe that marriages should last forever.					
29 My spouse and I remain married because we value the institution of marriage.					
30 I believe that marriage is for life regardless of what happens.					
31 A divorce would ruin my reputation.					
32 I have to stay married to my spouse or else my family will think badly of me.					
33 I was raised to believe that once one gets married, one doesn't get divorced, no matter how unsatisfying the marriage may be.					
34 It would be humiliating if my spouse and I divorced.					
35 Even if I wanted to, it would be impossible for me to leave my spouse.					
36 I would not be embarrassed to get a divorce.					
37 My family would strongly disapprove if I divorced my spouse.					
38 I've spent so much money on my relationship with my spouse that I could never divorce him or her.					

Supplementary Figure 3. The Dimension of Commitment Inventory (DCI)

Items	Strongly disagree	Disagree	Neither Disagree Nor Agree	Agree	Strongly agree
39 My friends would disapprove if I ended my marriage.					
40 If I was desperate for a divorce, I would find it a relief.					
41 I don't think I could handle the shame of being divorced.					
42 It would be shameful if my spouse and I divorced or separated.					
43 I could never leave my spouse; I have too much invested in him or her.					
44 I am afraid that if I leave my spouse, I will be punished					
45 It would be particularly hard on my family and friends if my spouse and I divorced.					

Supplementary Figure 4. The Dimension of Commitment Inventory (DCI)

Questionnaire 2

Instructions: There are many choices in married life, which represent the corresponding frequency and degree of sacrifices. The following 35 items simulate the possible situations in life that require sacrifice. Please read each sentence carefully and make two choices according to your real situation. Choice 1: In the past year, how often did you do this behavior; Choice 2: How much effort this behavior meant to you. There is no right or wrong answer. Thanks!

Items	Frequency					Degree/Cost of sacrifices				
	Nev-er	Ra-rely	So-metimes	Oft-en	A-lways	Not at all	Small	Mo-derate	Large	Extrem-ely high level
1 I attend my spouse's social events or get together with his/her family at his/her request.										
2 I canceled/changed my scheduled plans to be with my spouse if he/she wanted me to										
3 I attended events that my spouse was interested in, even if I was not interested in going.										
4 In order to accompany my spouse, I give up the activities I am interested in.										
5 I do my spouse a favor.										
6 In order to help my spouse, I do housework or follow his/her orders.										
7 In order to be with my spouse, I gave up doing my own job or doing housework.										
8 I help my spouse with his/her work or housework.										
9 When my spouse is sick or unwell, I sacrifice my time to care for him/her.										
10 I give to maintain my spouse's health or make him/her healthier. (e.g., eating healthy for him, not smoking in front of her, etc.)										

Supplementary Figure 5. Couples Sacrifice Behavior Scale (CSBS) (self-assessment)

11 I should stay with my spouse's family with his/her family.										
12 I limited time spent with my extended family to be with my spouse if he/she wanted me to.										
13 Even if my spouse is talking about something I'm not interested in, I still pay attention.										
14 I will do things in my spouse's way, not in my own way, in order to make him/her happy.										
15 I buy gifts for my spouse.										
16 I changed my spending habits to benefit my spouse.										
17 I changed the way I interacted with the opposite sex to please my spouse.										
18 I change my appearance (e.g., hairstyle, clothes, etc.) to please my spouse.										
19 I have sex with my spouse to please him/her.										
20 I was willing not to push for sex when my spouse did not want to.										
21 I express my love to my spouse to please him/her.										
22 Even if it is difficult, I will still meet the demands of my spouse.										
23 I do something for others (e.g., for a child, his/her family, or a neighbor) at the request of my spouse.										
24 When there is an inconsistency in making decisions, I will compromise in order to fulfill my spouse.										
25 I give up control of things and let my spouse have the final say.										

Supplementary Figure 6. Couples Sacrifice Behavior Scale (CSBS) (self-assessment)

26 I changed myself to adapt to my spouse's life, hygiene and other habits.										
27 I sacrificed my health to adapt to the lifestyle of my spouse.										
28 I change the way I see things in order to be in line with my spouse.										
29 I interrupt or adjust my original career development for the sake of my spouse.										
30 No matter how much I want to say it, when my spouse doesn't want to hear it, I don't say it anymore.										
31 I decreased the amount of time spent with friends for the sake of my spouse's needs.										
32 I give my spouse what we both like.										
33 I take on things that neither of us like to do.										
34 I adjust my behavior at home to match my spouse's work or rest.										
35 I changed the way I said something to my spouse for the sake of my spouse's feelings.										

Supplementary Figure 7. Couples Sacrifice Behavior Scale (CSBS) (self-assessment)

Questionnaire 3

Instructions: There are many choices in married life, which represent the corresponding frequency and degree/cost of sacrifices. The following 35 items simulate the possible situations in life that need to be sacrifice. Please read each sentence carefully, make choices according to what you feel about your spouse's situation. There is no right or wrong answer. There is also no need to think too much, just make two choices based on the real situation in your impression. Choice 1: In the past year, how often did you feel that your spouse did the following actions; Choice 2: How much do you think this behavior meant to your spouse. There is no right or wrong answer. Thanks!

Items	Perceived Frequency					Perceived Degree/Cost of Sacrifices				
	Ne-ver	Rar-ely	Some-times	Often	Alw-ays	Not at all	Small	Mode-rate	Large	Extr-emely high level
1 My spouse attends my social events or is with my family at my request.										
2 My spouse cancels or changes his original plan in order to join me.										
3 My spouse participates in activities that interest me, even if he/she is not interested.										
4 In order to accompany me, my spouse gave up the activities he was interested in.										
5 My spouse does me a favor.										
6 To help me, my spouse handles housework, or obeys me.										
7 My spouse gave up doing his own job or doing housework in order to be with me.										
8 My spouse helps me with my work or household chores.										

Supplementary Figure 8. Couples Sacrifice Behavior Scale (CSBS) (assessment of spouse)

9 My spouse sacrifices his time to take care of me when I am sick or unwell.										
10 My spouse does something to keep me healthy or make me healthier. (e.g., eating healthy for me, not smoking in front of me, etc.)										
11 My spouse stays with my family as I wish.										
12 My spouse shortens the time spent with his/her family in order to be with me.										
13 Even when I'm talking about something my spouse is not interested in, he/she pays attention.										
14 My spouse will do things in my way, not his/her own, in order to make me happy.										
15 My spouse buys me gifts.										
16 My spouse changes his/her own consumption habits for me										
17 My spouse changes the way he/she interacts with the opposite sex to please me.										
18 My spouse changes his appearance (e.g., hairstyle, clothes, etc.) to please me.										
19 My spouse would have sex with me to please me.										
20 When I don't want to have sex, my spouse doesn't force me anymore.										
21 My spouse expresses love to me in order to please me.										

Supplementary Figure 9. Couples Sacrifice Behavior Scale (CSBS) (assessment of spouse)

22 Even if it is difficult, my spouse will meet my demands.										
23 My spouse does something for others at my request (e.g., for the children, his/her family or neighbors).										
24 When there is disagreement in making a decision, my spouse will compromise to make it work for me.										
25 My spouse gave up control over things and let me have the final say.										
26 My spouse changes himself to suit my life, hygiene and other habits.										
27 My spouse sacrifices her health to fit my lifestyle.										
28 My spouse changes the way he sees things in order to be in line with me.										
29 My spouse interrupts or adjusts his original career development for me.										
30 No matter how much my spouse wants to say, when I don't want to listen, he/she doesn't say it.										
31 My spouse reduces contact with some of my friends at my request.										
32 My spouse gives me the things we both like.										
33 My spouse does things that neither of us likes to do.										
34 My spouse adjusts his behavior at home to match my work or rest.										
35 My spouse adjusts his expression for my sake.										

Supplementary Figure 10. Couples Sacrifice Behavior Scale (CSBS) (assessment of spouse)

Questionnaire 4

Instructions: Married life is a process full of feelings and experiences. The following 10 sentences describe some of your feelings in marriage. Please mark "√" on the corresponding item that best matches you after each sentence according to your actual situation. . I hope you can fill it out truthfully, do not seek the opinions of others, and complete it independently. The "we" in the entry refers to you and your spouse. There is no right or wrong answer. Thanks!

Items	Really not true	Probably not	Neither Disagree Nor Agree	Maybe	Really true
1 I don't like my spouse's character and personal habits.					
2 I am very satisfied with the responsibilities of both spouse in the marriage.					
3 I am not satisfied with the communication between husband and wife, and my spouse does not understand me.					
4 I am very satisfied with the way we make decisions and resolve conflicts.					
5 I am not satisfied with our economic status and the way economic affairs are decided.					
6 I am very satisfied with our spare time and the time we spend as a couple.					
7 I am very satisfied with how we express our emotions and sexual matters between husband and wife.					
8 I am not satisfied with the division of responsibilities as a parent.					
9 I am not satisfied with our relationship with both parents and friends.					
10 I feel good about our values.					

Supplementary Figure 11. Evaluating and Nurturing Relationship Issues, Communication, Happiness (ENRICH)

Source

Adams, J. M., & Jones, W. H. (1997). The conceptualization of marital commitment: An integrative analysis. *Journal of Personality and Social Psychology*, 72(5), 1177 – 1196. https://doi.org/10.1037/0022-3514.72.5.1177

Li, T. (2006). *The psychological research of marital commitment* [Doctoral Dissertation, East China Normal University]. China National Knowledge Infrastructure. https://kns.cnki.net/kcms/detail/detail.aspx?dbname=CDFD2006&filename=2006124752.nh&dbcode=CDFD

Cao, H., Fang, X., Fine, M. A., Ju, X., Lan, J., & Zhou, N. (2016). Sacrifice, commitment, and marital quality in the early years of Chinese marriage: An actor–partner interdependence moderation model. *Journal of Social and Personal Relationships*, 34(7), 1122-1144. https://doi.org/10.1177/0265407516670041

Lan, J. (2010). *The Relationship between Sacrifice Motivation, Sacrifice Behavior and Marital Quality in Couples* [Master's Thesis, Beijing Normal University]. China National Knowledge Infrastructure.

Mao, H. (2019). *The practical applications of SATIR growth group in improving marriage quality-taking couples growth group who have been married for 2-8 years as an example* [Master's Thesis, Guangzhou University]. China National Knowledge Infrastructure. https://kns.cnki.net/kcms/detail/detail.aspx?dbname=CMFD2020&filename=1019615042.nh&dbcode=CMFD

Supplementary Figure 12. Source
